# Study protocol for a pragmatic cluster randomized controlled trial to improve dietary diversity and physical fitness among older people who live at home (the “ALAPAGE study”)

**DOI:** 10.1186/s12877-022-03260-8

**Published:** 2022-08-04

**Authors:** Aurélie Bocquier, Anne-Fleur Jacquemot, Christophe Dubois, Hélène Tréhard, Chloé Cogordan, Gwenaëlle Maradan, Sébastien Cortaredona, Lisa Fressard, Bérengère Davin-Casalena, Agnès Vinet, Pierre Verger, Nicole Darmon, on behalf of the ALAPAGE Study Group, Valérie Arquier, Guillaume Briclot, Rachel Chamla, Florence Cousson-Gélie, Sarah Danthony, Karin Delrieu, Julie Dessirier, Catherine Féart, Christine Fusinati, Rozenn Gazan, Mélissa Gibert, Valérie Lamiraud, Matthieu Maillot, Dolorès Nadal, Christelle Trotta, Eric O. Verger, Valérie Viriot

**Affiliations:** 1 ORS PACA, Observatoire Régional de la Santé Provence-Alpes-Côte d’Azur, Marseille, France; 2 Université de Lorraine, APEMAC, F-54000 Nancy, France; 3 Bordeaux Population Health Research Center, University of Bordeaux, Inserm, UMR 1219, F-33000 Bordeaux, France; 4 Trophis, 13170 Les Pennes Mirabeau, France; 5 Aix Marseille Univ, IRD, INSERM, SESSTIM, Aix Marseille Institute of Public Health, ISSPAM, Marseille, France; 6 Aix Marseille Univ, IRD, AP-HM, SSA, VITROME, Marseille, France; 7 IHU-Méditerranée Infection, Marseille, France; 8 Avignon Université, UPR EA4278, F-84000 Avignon, France; 9 MoISA, Université de Montpellier, CIHEAM-IAMM, CIRAD, INRAE, Institut Agro, IRD, Montpellier, France

**Keywords:** Healthy ageing, Elderly people, Nutrition, Dietary diversity, Physical activity, Lifestyle integrated functional exercise, Quality of life, Health education, Cluster randomized controlled trial

## Abstract

**Background:**

Diet and physical activity are key components of healthy aging. Current interventions that promote healthy eating and physical activity among the elderly have limitations and evidence of French interventions’ effectiveness is lacking. We aim to assess (i) the effectiveness of a combined diet/physical activity intervention (the “ALAPAGE” program) on older peoples’ eating behaviors, physical activity and fitness levels, quality of life, and feelings of loneliness; (ii) the intervention’s process and (iii) its cost effectiveness.

**Methods:**

We performed a pragmatic cluster randomized controlled trial with two parallel arms (2:1 ratio) among people ≥60 years old who live at home in southeastern France. A cluster consists of 10 people participating in a “workshop” (i.e., a collective intervention conducted at a local organization). We aim to include 45 workshops randomized into two groups: the intervention group (including 30 workshops) in the ALAPAGE program; and the waiting-list control group (including 15 workshops). Participants (expected total sample size: 450) will be recruited through both local organizations’ usual practices and an innovative active recruitment strategy that targets hard-to-reach people. We developed the ALAPAGE program based on existing workshops, combining a participatory and a theory-based approach. It includes a 7-week period with weekly collective sessions supported by a dietician and/or an adapted physical activity professional, followed by a 12-week period of post-session activities without professional supervision. Primary outcomes are dietary diversity (calculated using two 24-hour diet recalls and one Food Frequency Questionnaire) and lower-limb muscle strength (assessed by the 30-second chair stand test from the Senior Fitness Test battery). Secondary outcomes include consumption frequencies of main food groups and water/hot drinks, other physical fitness measures, overall level of physical activity, quality of life, and feelings of loneliness. Outcomes are assessed before the intervention, at 6 weeks and 3 months later. The process evaluation assesses the fidelity, dose, and reach of the intervention as its causal mechanisms (quantitative and qualitative data).

**Discussion:**

This study aims to improve healthy aging while limiting social inequalities. We developed and evaluated the ALAPAGE program in partnership with major healthy aging organizations, providing a unique opportunity to expand its reach.

**Trial registration:**

ClinicalTrials.gov Identifier: NCT05140330, December 1, 2021.

Protocol version: Version 3.0 (November 5, 2021).

**Supplementary Information:**

The online version contains supplementary material available at 10.1186/s12877-022-03260-8.

## Background

### Relevance

Between 2015 and 2050, the proportion of the world’s population over 60 years old will increase from 12 to 22% (and from 25 to 34% in France) [1]. Older age is associated with a greater prevalence of chronic diseases, frailty, dependence, and associated healthcare costs [2–4]. This has led the United Nations General Assembly to declare 2021–2030 the “Decade of Healthy Ageing” which aims to optimize older peoples’ functional abilities (e.g., ability to meet one’s basic needs; to learn, grow, and make decisions; to build and maintain relationships; and to be mobile) [2]. The Decade of Health Ageing includes equity as one of its guiding principles, highlighting that some population groups may sometimes require more attention to ensure the greatest benefit to the least advantaged [5].

The World Health Organization identified nutrition and physical activity as key components that influence healthy ageing [6]. Healthy diets among the elderly may help to maintain autonomy and to increase activity limitation and limitation-free life expectancy: in particular, it has been associated with a reduced risk of physical frailty [7–10], activity limitation [11], decline in cognitive function, [12] and death [13]. Physical activity in older age also helps to maintain autonomy, reduces risks of diseases like coronary heart disease or diabetes, improves physical and mental health capacities, as well as quality of life and social outcomes (e.g., community involvement, maintenance of social networks) [6, 14]. Physical activity can especially help reduce social isolation and loneliness. These growing public health concerns have been made even more salient by the COVID-19 pandemic [15].

Several behavioral interventions promoting healthy eating among community-dwelling elderly people have been implemented worldwide. Most of them have included meal services and have targeted frail older people [16–19]. Other behavioral interventions that targeted broader populations of community-dwelling elderly people and included dietary educational interventions, found either no significant effect [20, 21] or positive impacts on diet, nutritional status and other outcomes like self-efficacy or social support [22–27]. Some of the effective interventions [22, 25, 26] originally aimed at improving dietary diversity among older people: eating a wide range of foods is positively associated with nutritional adequacy in diets and contributes to the prevention of frailty [28–32]. Many interventions which target older peoples’ physical activity levels have also been implemented, most frequently, to reduce the risk of falls [33]. These programs focused on improving posture, balance, and walking. Effective programs usually lasted 3 months (three times a week); however, some shorter programs (lasting five to eight weeks) were also found to improve balance and mobility issues [34–36]. The Australian Lifestyle-integrated Functional Exercise (LiFE) program, which combines both balance and strength training, merits particular interest. This program is an innovative intervention based on a new dual-tasking approach where exercises are included into the elderly’s usual daily activities in their everyday lives [37]. This program showed greater adherence than traditionally structured programs and had positive impacts on the risk of falls and the maintenance of functional capacities [37].

However, few studies have assessed the effectiveness of combined programs that target both dietary habits and physical activity among older adults who live at home (e.g., [25, 27]). These studies used traditional recruitment strategies and also did not make concerted efforts to recruit hard-to-reach older populations (e.g., those who live in deprived areas), who are known to participate infrequently in health promotion interventions [38]. Active recruitment strategies (i.e., direct, face-to-face contact) could be particularly effective in improving participation of hard-to-reach older people, but further evidence is needed [38, 39]. Finally, as part of the French national strategy for healthy aging [40], several national and regional stakeholders (e.g., the French Public Health Agency, regional health agencies, retirement funds, associations involved in health promotion) have been supporting health promotion interventions that target older peoples’ diet and physical activity for many years (most frequently as collective prevention workshops). However, no study to date has been conducted to assess their effectiveness [41].

### Objectives

In this context, we designed the ALAPAGE study to assess:

1. the effectiveness of a combined diet and physical activity intervention (the “ALAPAGE program”) on older peoples’ dietary diversity and eating behaviors; physical activity and fitness; and quality of life and feelings of loneliness (effectiveness evaluation);
2. the fidelity, dose, and reach of the intervention as well as the mechanisms by which the intervention may modify outcomes (process evaluation);
3. the cost effectiveness of the intervention (economic evaluation).

The present article describes the ALAPAGE study protocol in conformance to SPIRIT’s (Standard Protocol Items: Recommendations for Interventional Trials) 2013 statement [42] (see completed SPIRIT checklist in Additional file 1).

## Methods

### Study design and setting

The ALAPAGE study is a pragmatic cluster randomized controlled trial (cRCT) [43] with two parallel arms using a 2:1 ratio. It is performed among older people who live at home in southeastern France (intervention period: January 2022–October 2023). A cluster consists of approximately 10 people participating in a “workshop”, which is defined as a collective intervention that is conducted on the premises of local organizations (e.g., municipalities, social/community centers). A total of 45 workshops are randomized into two groups: (i) the intervention group (corresponding to 30 workshops), who benefits from the ALAPAGE program; and (ii) the control group (corresponding to 15 workshops), who will only benefit from the ALAPAGE program after the completion of the study (waiting-list control group).

**Fig. 1:**
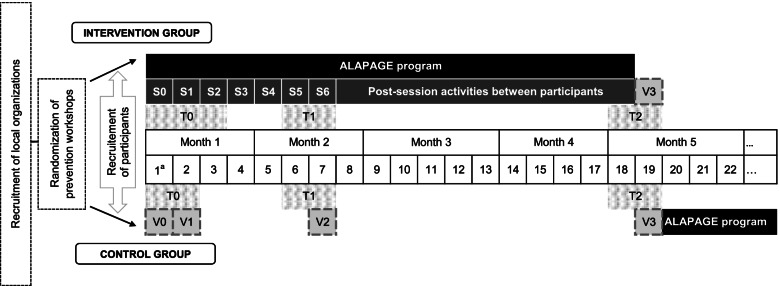
Schematic overview of the ALAPAGE study. S0-S6: diet and physical activity sessions of the ALAPAGE program; T0-T2: evaluation time points; V0-V3: measurement visits ^a^Week number

The complete study duration for participants is four and a half months (19 weeks; see Fig. 1 for an overview of the study). Participants in the control group have measurement visits, but no intervention visits before benefiting from the ALAPAGE program.

The ALAPAGE study design was tested in a pilot study between September and February 2022 and was based on two intervention and one control workshops. This pilot study led us to make several modifications to the intervention (e.g., the increase of the sessions’ duration from 2 to 2.5 hours; the revision of the training content/duration that professionals undertook before the intervention; the improvements to the intervention tools), the data collection procedures (e.g., the addition/removal of items in self-administered questionnaires) and research documentation (e.g., the revision of the information sheets to make them more readable).

### Participants and recruitment procedure

We aim to recruit 450 adults, age 60 years old or over who live at home, and split them across 45 workshops (30 intervention and 15 control workshops) with on average 10 participants in each (see details in paragraph “Sample size calculation”).

#### Selection of local organizations and allocation of workshops

First, we looked for local organizations who would volunteer to host the workshops as part of the ALAPAGE study. The research team, with support from its operational partners (e.g., the regional retirement fund) launched the call for applications to invite local organizations to participate in the study. Local organizations who were interested in the study filled out an information sheet (requesting information such as location, number of older people in their active file, experience in organizing prevention workshops, period of availability to organize one or more workshops, availability of a “point-of-contact” person to manage the logistical aspects of the workshops, the provision and storage of materials, etc.) and returned it to the research team. A total of 45 local organizations submitted proposals between June 1, 2021 and July 16, 2021. The research team and its operational partners then selected 25 out of the 45 organizations to host a total of 45 workshops. The selections were based on a reasoned choice and operational criteria (e.g., availability, materials, and staff resources).

We performed a block randomization with the use of computer-generated randomization lists. We created four time-based blocks (start date of the workshop); each observed a 2:1 allocation ratio. We did this to take into account the seasonality of both diet and physical activity behaviors in addition to any potential disruptions due to the COVID-19 pandemic (block 1: 12 workshops between January 2022–April 2022; block 2: 12 workshops between May 2022–October 2022; block 3: 15 workshops between November 2022–April 2023; block 4: 6 workshops between May 2023–June 2023).

#### Recruitment of participants

Participants are then recruited by one of two methods:

1. Recruited according to the usual practices of local organizations (e.g., announcements and advertisements within the organization, local newspapers, public posters, leaflets). The inclusion criteria are: age ≥ 60 years, who live at home, have health insurance coverage and are able to read and write in French. The non-inclusion criteria are: receiving allocation for dependence (i.e., belonging to one of the fourth-highest groups of the AGGIR grid, a six-level scale used in France to measure the independency levels of elderly people [44, 45]), being under guardianship, and having participated in a previous prevention workshop on diet or physical activity within the last 2 years. These criteria aim to target individuals: who, according to the operational partners’ usual practices within the field of healthy aging, are suitable for the preventive intervention program; who meet the research requirements (e.g., are able to give free and informed consent and to complete self-administered questionnaires) and who meet the regulatory requirements (health insurance).
2. Recruited using an active recruitment strategy, which was previously designed and pilot-tested by our research team, that targets hard-to-reach people [46]. In sum, this strategy aims to increase the participation of socioeconomically disadvantaged people and/or socially isolated older people in health-prevention programs on diet and physical activity by the following four steps: (i) identification in the retirement fund database of targeted older people; (ii) invitation letter by mail; (iii) telephone call by a social worker; (iv) home visit by the same social worker. The inclusion criteria are: 60–80 years old who live at home in the municipality where the prevention workshop will take place, receive allocation for economically deprived and/or socially isolated older people, have a telephone number recorded in the retirement fund database and be able to read and write in French. The non-inclusion criteria are the same as the aforementioned for people recruited by usual pathways. For each workshop of 10 participants, we aim to recruit 3 participants through this active strategy.

Staff from local organizations --for (1)--, or social workers --for (2)--, relay information about the study both orally and in writing and prescreen participants for eligibility criteria. During the inclusion session/visit (S0 and V0 for the intervention and control groups respectively, see Fig. 1), the dietician reads the written information aloud to the participants, verifies eligibility criteria and asks for informed consent in writing.

### Interventions

#### Intervention group

The intervention (the ALAPAGE program that means “up to date” in French) was designed based on a diagnostic study that we performed in 2016–2017 to examine strengths, limitations, ways of improving existing prevention workshops on diet and physical activity for the elderly in France [41], and on the scientific literature. In particular, this diagnostic study highlighted the need to improve the attractiveness of these workshops to the elderly, the participation of socioeconomically-disadvantaged and socially-isolated people, and participation maintenance throughout the workshop sessions. We optimized the content of existing workshops by involving dieticians and APA professionals as part of the participation process, and used intervention mapping as a guide [47]. In sum, based on the Theory of Planned Behavior [48], the ALAPAGE program aims to improve participants’ attitudes, perceived norms, perceived self-control, and intention to improve diet and physical activity. Moreover, to increase the probability of behavior change and reduce the intention-behavior gap, we introduced additional behavior change techniques like setting goals, planning actions, using feedback and monitoring, and reviewing goals [49].

Each intervention workshop includes (i) a 7-week intervention period with weekly collective sessions supported by a dietician and/or a qualified APA professional (one introductory session, 4 sessions on diet, and 2 sessions on physical activity, each 2.5 hours in duration); followed by (ii) a 12-week intervention period that consists of a post-sessional program of activities among participants without the supervision of a professional (see Table 1 for more details). As part of this pragmatic trial, professionals (e.g., dieticians, APA professionals and staff from local organizations) have some flexibility in the implementation of the ALAPAGE program in order to take into account for “real-life” constraints (e.g., they can opt for a two-week interval between sessions instead of a one-week interval) [43]. Compared to existing workshops, the ALAPAGE program newly addresses the following themes: dietary diversity, nutritional profiles, budget (healthy eating on a budget), sustainable diet and, regarding physical activity, balance, flexibility, strength and aerobic exercises incorporated into everyday tasks [37], and a 10-minute routine exercise (see Table 1 for more details). The ALAPAGE program uses innovative tools developed as part of the participation process and involved dieticians, APA professionals, and elderly people.

**Table 1:** Overview of the ALAPAGE program^a^

Content of the ALAPAGE program	Supervisor(s)
**S0: inclusion/introductive session**	
Presentation of the supervisors, participants, and workshop program; quiz on diet and physical activity; example of completion of a weekly notebook for physical activity self-reporting and provision of one^b^.	Dietician + APA professional
**Inter-session: physical activity self-monitoring at home**	
**S1: introduction to diet diversity**	
24-hour diet recall; discussion on participants’ beliefs on diet; game ‘11 food families’ (cards); analyses of one’s own diet using the ALAPAGE grid of dietary diversity; distribution of pedometers.	Dietician
**Inter-session: physical activity self-monitoring at home**	
**S2: physical activity**	
Feedback on inter-sessions; warming; Senior Fitness Test battery and static balance test; learning of exercises that can be introduced in daily life activities (dual-tasking) and of a 10-minute routine exercise; selection of one physical quality to improve.	APA professional
**Inter-session: physical activity everyday tasks and self-monitoring at home**	
**S3: improving diet diversity by choosing foods with good nutritional quality**	
Feedback on inter-sessions; game on nutritional profiles; information on diseases prevention and local resources regarding diseases management; improving one’s own diet diversity and setting a personalized objective.	Dietician
**Inter-session: physical activity everyday tasks and self-monitoring at home**	
**S4: introduction to sustainable diet**	
Feedback on personalized objectives and the inter-session; photo language about sustainable diet; preparation of the post-session program of activities between participants; revision of the personalized objective.	Dietician
**Inter-session: physical activity everyday tasks and self-monitoring at home**	
**S5: diversifying pleasures to eat better at a ‘good’ price**	
24-hour diet recall; feedback on personalized objectives; activity ‘Pleasures and budget’ using same foodstuffs from different brand; preparation of the post-session program; analyses of one’s own diet using the ALAPAGE grid of dietary diversity; revision of the personalized objective.	Dietician
**Inter-session: physical activity everyday tasks and self-monitoring at home**	
**S6: physical activity**	
Feedback on inter-sessions; static balance test and comparison with results from S2; warming; 45 min physical activity training; preparation of the post-session program.	APA professional
**Post-session program of activities between participants (e.g., walking).**	

*APA* adapted physical activity, *S0-S6* diet and physical activity sessions of the ALAPAGE program

^a^Each session lasted 2 h30

^b^A weekly notebook for physical activity self-reporting is also provided to participants in each following session from S1 to S5

#### Control group

Participants in the control group will receive no intervention and will only benefit from the ALAPAGE program at the end of the research study. In order to encourage retention, however, the inclusion visit (V0) includes light refreshments and the measurement visit (V2) includes an interesting one-hour activity on waste recycling (see details on the content of the control group measurement visits in Additional file 2).

### Outcomes

#### Effectiveness evaluation

Primary and secondary outcomes, with few exceptions, are assessed before the intervention (“T0”), at 6 weeks (“T1” corresponding to the end of ALAPAGE program’s session period) and then 3 months later (“T2”) (see Table 2 for an overview of outcomes and data time points).

**Table 2:** Overview of outcomes and time point of data collection

TIME POINT		Week 1	Week 2	Week 3	Week 4	Week 5	Week 6	Week 7	Week 8	Week 18	Week 19
	I	S0	S1	S2	S3	S4	S5	S6	Home	Home	V3
		T0					T1			T2	
	C	V0	V1				Home	V2		Home	V3
		T0					T1			T2	
**ASSESSMENTS**											
***Primary outcomes***											
Dietary diversity (2 24-hour recalls + 1 FFQ)	I	X	X				X	X		X	X
	C	X	X				X	X		X	X
Lower limb muscle strength (Senior Fitness Test)^a^	I			X							X
	C		X								X
***Secondary outcomes effectiveness evaluation***											
Physical fitness (upper body strength, aerobic endurance, flexibility, dynamic balance) (Senior Fitness Test)^a^	I			X							X
	C		X								X
Static balance	I			X				X			X
	C		X					X			X
Number of steps during one week (pedometer)	I			X			X			X	
	C	X					X			X	
Self-reported global level of physical activity (QAPPA questionnaire)	I			X				X			X
	C		X					X			X
Frequency of consumption of 11 food families and of water/hot drinks (2 24-hour recalls + 1 FFQ)	I	X	X				X	X		X	X
	C	X	X				X	X		X	X
Quality of life (SF-12) + Feeling of loneliness (item from the FRAGIRE grid)	I		X				X				X
	C	X						X			X
***Secondary outcomes process evaluation***											
Fidelity, dose, reach of the intervention (attendance lists, information sheets completed by the local organizations, dieticians and APA professionals after each session)	I	X	X	X	X	X	X	X			
Fidelity, dose of the intervention (questionnaire on self-reported exercises in daily activities, participation in post-session activities between participants)	I							X			X
Mechanisms of the intervention (questionnaire on beliefs, attitudes, intention towards physical activity)	I			X				X			X
***Cost-effectiveness evaluation***											
Costs	I	X	X	X	X	X	X	X	X		
	C	X	X	X	X	X	X	X	X		

*APA* adapted physical activity, *C* control group, *FFQ* Food Frequency Questionnaire, *I* intervention group, *S0-S6* diet and physical activity sessions of the ALAPAGE program, *T0-T2* evaluation time points, *V0-V3* measurement visits

^a^Lower limb muscle strength and other physical functions measured using the Senior Fitness Test battery are assessed at T0 and T2 but not at T1 because the interval time between T0 and T1 is too short to expect a significant impact of the intervention on these outcomes at T1

##### Primary outcomes

*Dietary diversity* is assessed using the Diversity ALAPAGE Score (DAS). Based on dietary diversity scores developed abroad [50–52], we developed the DAS using individuals’ consumption habits of 20 food categories (see Additional file 3 for more details). Individuals must complete two 24-hour diet recalls (for foods that they usually eat every day, such as fruits, vegetables, meat excluding poultry and sweetened products) and one Food Frequency Questionnaire (FFQ) (for foods that they consume less frequently, such as eggs, legumes, nuts, fatty fish, see Additional file 4). Each consumption earns positive or negative points according to the nutritional quality of its food category. The DAS for one individual is calculated by adding all attributed points. A higher DAS means a greater diversity in diet. We found that DAS was positively associated with the quality of diet among a representative sample of French people age 60 years or older [53], and that a low DAS was associated with an increased death risk among a cohort of older French people who were followed over 15 years [54].

*Lower-limb muscle strength* is assessed using the 30s Chair Stand Test from the Senior Fitness Test battery (adapted in French) [55]. Participants repeatedly stand up from and sit down on a chair for 30 seconds and an APA professional records the number of complete stands.

##### Secondary outcomes

*Consumption frequencies of main food groups and water/hot drinks* are assessed using data from two 24-hour diet recalls and one FFQ.

*Physical fitness* is assessed using the French version of the Senior Fitness Test battery [55]. Besides the 30s Chair Stand Test which indicates lower-body strength (see primary outcomes), it also includes: (i) the Arm Curl Test (upper-body strength); (ii) the 2-minute Step Test (aerobic endurance); (iii) the Chair Sit and Reach Test (lower-body flexibility); (iv) the Back Scratch Test (upper-body flexibility); and (v) the Get Up and Go Test (dynamic balance). Additionally, the Open-Eye Stand on Dominant Foot Test assesses static balance. According to the range of scores [56], there are four levels for each physical parameter: low, below average, to be maintained, and good.

*Overall level of physical activity* is assessed by (i) the number of steps recorded with a pedometer (Yamax Power-Walker EX_™_-210) during 1 week and reported by the participant on a weekly notebook provided by the APA professional; (ii) the *Questionnaire d’activité physique pour personnes âgées* (QAPPA) self-reported physical activity questionnaire for the elderly [57]. This 7-day recall questionnaire includes 4 items on moderate and intense physical activity. Scores are calculated using the number of minutes of each type of activity reported in metabolic equivalent per week and individuals are then classified into three activity levels (low, moderate, or high).

*Quality of life* is measured by the SF-12 V1 Health Survey questionnaire [58] which includes 12 questions corresponding to the following eight domains: 1) Limitations in physical activities because of health problems; 2) Limitations in social activities because of physical or emotional problems; 3) Limitations in usual role activities because of physical health problems; 4) Bodily pain; 5) General mental health; 6) Limitations in usual role activities because of emotional problems; 7) Vitality; and 8) General health perceptions.

*Feelings of loneliness* are assessed using one item of the environmental dimension of the FRAGIRE tool for assessing an older person’s risk for frailty [59]: “Do you feel lonely or abandoned?” (*Not at all*, *A little*, *Quite a bit, A lot).*

#### Process evaluation

Intervention fidelity, dose, and reach are assessed using data obtained from attendance lists, information sheets completed by local organizations, information sheets completed by dieticians and APA professionals after each session, and self-administered questionnaires that are completed by participants during T1 and T2 (e.g., self-reported exercises from everyday tasks; participation in post-session activities between participants, see Additional file 5) [60]. To ensure fidelity, and to make sure that the program is transferable, we will also document how the actors who are involved in the program interact [61], in comparison with planned interactions.

To explore how the intervention might modify outcomes, we will analyze data from the self-administered questionnaires to be completed by participants at T0, T1 and T2, especially from the questions that assess the major constructs of the Theory of Planned Behavior [48] (beliefs, attitudes, and intention, see Additional file 5). In the Theory of Planned Behavior questionnaires, the behavior of interest must be clearly defined in terms of target, action, context, and time elements. We measured the beliefs, attitudes, and intention of physical activity (“daily physical activity in the next 3 months”) [62], but found that the questions were not applicable to dietary diversity. We also conduct semi-structured individual interviews with approximately 20 participants from the intervention group, who accepted to be contacted again, to gain a better understanding of how/why participation in ALAPAGE workshops improves (or does not improve) eating behaviors, physical activity, and quality of life, as facilitators and barriers to behavior changes that participants encountered.

#### Cost-effectiveness evaluation

All unit costs (e.g., hourly cost for dieticians, APA professionals) and quantities required (e.g., total number of working hours) were listed and collected in advance from the local organizations who are responsible for the intervention and control workshops. These costs will then be compared to the effectiveness of the intervention (outcomes related to eating behaviors, physical activity, and Quality Adjusted Life Years, QALYs [63]) using the cost-effectiveness ratio (i.e., incremental costs divided by the incremental health benefits) [64].

#### Sample characterization

Sociodemographic data (e.g., age, sex, educational level, perceived financial situation, frequency of participation in social activities) as antecedents towards physical activity and history of falls are also collected through self-questionnaires (see Additional file 6).

### Data collection procedure

Data collection is performed on the premises of local organizations under the supervision of a dietician and/or an APA professional, except for: one 24-hour recall performed at home during week 5 for the control group and during week 18 for both groups; and the number of steps during 1 week recorded at home three times for each group (Table 2).

Face-to-face interviews with participants are performed by researchers, who have significant qualitative research experience, in the month following T2 at the local organization or at home depending on the participants’ preference.

All study participants will be financially compensated (the intervention group will receive a 15 € voucher; the control group will receive two 15 € vouchers, the second voucher compensating for the additional participation in the measurement visit).

Expected and non-expected serious adverse events and other unintended effects of the intervention (i.e., those that require hospitalization, result in a significant or long-lasting disease/disability, or death) are collected and immediately reported to the research team by the sessions’ dieticians and APA professionals.

Prior to the commencement of the study, dieticians and APA professionals participate in training sessions that are led by research team members (including by one person who is certified in Good Clinical Practices [GCP]) and includes (i) a two-hour video conference that presents the study and its data circuit; (ii) a half-day session on research issues (e.g., clinical practices, information, consent, notification of adverse events); and a further one and a half day session on the study’s content and visits (e.g., program, objectives, tools).

### Sample size calculation

Sample size calculations are based on the (i) research team’s preliminary results on dietary diversity and the development of the DAS [53], and the research team’s consensus on the intervention’s effect size of + 1 standard deviation between T0 and T2; (ii) impact on older people’s lower-limb muscle strength from a previous intervention that showed similarities with the exercises proposed in the ALAPAGE program [65]. We performed power calculations using simulations (10,000 samples per simulation, see Additional file 7 for more details) assuming α = 0.05, similar characteristics of participants in both groups, a 30% dropout rate between T0 and T2, and an intra-cluster correlation of 0.03 [22, 66]. Based on these simulations, we intend to enroll 300 participants in the intervention group (i.e., 30 workshops with 10 participants in each) and 150 participants in the control group (15 workshops with 10 participants in each); this provides a 95% power for both primary outcomes.

### Data management

The participants are each given a unique identification code (without any personal information that could allow for their identification). During the study period, all of the collected data is stored in a locked cabinet at the local organizations, according to a procedure compliant with GCP guidelines. When the study is complete, the collected data will be securely transmitted to the research team’s logistic department, who will create the databases. The collected data will be stored according to standards for archiving research materials.

Leaders of the research team who are involved in the ALAPAGE study and who have signed a consortium agreement will have access to the final study dataset.

### Data analysis

All statistical analyses will be performed using SAS 9.4 (SAS Institute Inc., Cary, NC, USA). A α of 0.05 will be used to determine statistical significance.

First, descriptive statistics (mean ± standard deviation, median, and frequency distribution) will be carried out to describe baseline characteristics (sociodemographic, life conditions, primary and secondary outcomes) of participants in both groups. To compare the baseline characteristics (age, gender, life conditions) between the two groups, a one-way ANOVA or a Kruskal-Wallis test will be used for continuous variables and a Chi-square or a Fisher’s exact test will be used for categorical variables.

As part of the effectiveness evaluation, linear or logistic (depending on the nature of the dependent variable) mixed models will be carried out to study the impact of the intervention on primary and secondary outcomes, while taking into account the repeated nature of the data and the intra-cluster correlation. Time, group and their interaction will be defined as fixed factors (see Additional file 7 for more details). If imbalances occur between the groups, the baseline values will be added as covariates in the models. Missing data will be inspected, and, if appropriate, will be handled using multiple imputation.

As part of the process evaluation, we plan to perform mediation analyses to verify whether beliefs, attitudes, and intention are pathways by which the intervention impacts physical activity. Furthermore, we plan to perform moderating analyses to test whether the effect of the intervention varies according to some characteristics (in particular, type of recruitment, antecedents of physical activity or history of falls, and baseline dietary diversity).

As part of the process evaluation, qualitative data from participant interviews of the intervention group will be transcribed and analyzed using a data-driven inductive approach to explore how the intervention may have resulted in behavioral changes.

### Dissemination policy

The results of the study will be communicated by the research team to the local organizations who participated in the study, the professional sponsors who are involved in healthy ageing and also the public (e.g., via the websites of the retirement fund and other operational partners). The study will result in several publications in peer-reviewed journals.

## Discussion

The ALAPAGE study is a pragmatic cRCT which aims at assessing the effectiveness, process, and cost effectiveness of a combined diet and physical activity collective intervention among older French people who live at home. It is conducted by a multidisciplinary research team (epidemiology/public health, human nutrition, physical activity, health economics, social psychology) in close partnership with experienced operational partners in health promotion among elders.

This study has several strengths. First, the ALAPAGE program on diet and physical activity evaluated in this study is based on “real-life” interventions, i.e., collective prevention workshops that have been implemented by our operational partners for many years. Based on a diagnostic study, we optimized these workshops and tools using both a participatory approach involving dieticians, APA professionals and elderly people, and a theory-based approach [48, 49]. This development process is recommended to enhance the interventions’ fidelity, suitability to context, and effectiveness [67]. Secondly, this study includes an innovative active recruitment strategy to improve participation of hard-to-reach (i.e., socioeconomically-disadvantaged and socially-isolated) older people. It will thus contribute to creating greater equity in healthy ageing [5] and help prove the effectiveness of strategies aimed at identifying socially isolated/lonely people and also connect them to services [15]. Third, this cRCT includes a wide range of outcomes relating to eating behaviors, physical activity and fitness but also to social aspects like quality of life and feelings of loneliness. The methodology will also allow us to explore the causal mechanisms of the intervention and to understand how the ALAPAGE program – if effective, as we hypothesize -- improves the behaviors of participants [67].

We must also acknowledge some limitations. First, the recruitment of participants will take place after the randomization of workshops. This may lead to recruitment bias and a discrepancy in the characteristics of the participants in both the intervention and the control groups [68]. As part of this pragmatic cRCT, our operational partners did not find it feasible to recruit participants before the cluster randomization or by means of a blinded and independent person. To limit recruitment bias (e.g., participants in the intervention group are more interested in diet and physical activity issues and are thus more likely to change their behaviors than those in the control group), we will use a waiting-list control group design. Consequently, all participants are interested in participating in workshops on diet and physical activity. Second, due to feasibility constraints, the times points differ slightly between the intervention and control groups for some of the outcomes. However, we have done our best to limit these differences and to reconcile methodology requirements and practical feasibility. We hypothesize that this will have a limited impact on our results. Lastly, we cannot exclude differing attrition rates between the intervention and control groups that may lead to biased estimates of the intervention’s effects. In this instance, we plan to use appropriate econometric techniques (e.g., a Heckman sample selection correction model [69]) to correct for attrition bias [70].

The results of this study should help to implement public health interventions that are effective at improving dietary diversity, physical activity and fitness, and social outcomes among older people who live at home. They will contribute to the improvement of healthy aging while limiting social inequalities. The ALAPAGE program has been developed and will be evaluated in close relationship with major operational partners of healthy aging in France, thus providing a unique opportunity to expand its reach.

### Roles and responsibilities

The steering committee for the ALAPAGE study is comprised of the study’s scientific coordinator, the principal investigator (and representative of the study’s sponsor) and several of his team members who are responsible for the project’s management, representatives from operational partners (Carsat Sud-Est, ASEPT PACA, Mutualité Française Sud, Géront’ONord, SudEval and Trophis), and a number of representatives from the scientific committee. The steering committee meets regularly depending on the research’s needs, including reviewing the study’s progress and providing overall feedback to each of its members. It is also responsible for making any important decisions regarding the proper conduct of the study and compliance with the protocol. It verifies ethical compliance.

Finally, a scientific committee composed of experts in health psychology, nutritional epidemiology, adapted physical activity and health economics meet at least once a year (or more times if necessary) to validate methodological aspects of the study, provide guidance on the study’s conduct and the dissemination of results.

## Supplementary Information

**Additional file 1. **SPIRIT 2013 Checklist.

**Additional file 2. **Control group measurement visits’ content.

**Additional file 3. **Diversity ALAPAGE Score: calculation method.

**Additional file 4. **Food Frequency Questionnaire (FFQ).

**Additional file 5. **Self-administered questionnaires’ sections related to process evaluation.

**Additional file 6. **Self-administered questionnaires’ section related to participant’s characteristics.

**Additional file 7. **Sample size calculation.

## Data Availability

Not applicable.

## Abbreviations

APA: Adapted physical activity
cRCT: cluster randomized controlled trials
DAS: Diversity ALAPAGE Score
FFQ: Food Frequency Questionnaire
GCP: Good Clinical Practices
LiFE: Lifestyle-integrated Functional Exercise program
QALYs: Quality Adjusted Life Years
