# Supplementary material for: Study protocol for a pragmatic cluster randomized controlled trial to improve dietary diversity and physical fitness among older people who live at home (the “ALAPAGE study”)
Source: BMC Geriatr. 2022 Aug 4;22:643. doi: 10.1186/s12877-022-03260-8 (PMC9351201; doi:10.1186/s12877-022-03260-8)
Supplement: Supplementary file 2 — Additional file 2. Control group measurement visits’ content. [file 12877_2022_3260_MOESM2_ESM.docx]

**Additional file 2.** Control group measurement visits’ content^a^

| **Content of the visit** | **Supervisor** |
| --- | --- |
| **V0 (inclusion/introductive visit)** | |
| Presentation of the supervisors, participants, and visits program; information sheet and consent; 24-hour diet recall and self-administered questionnaire (including FFQ); refreshments; distribution of pedometers and explanations. | Dietician and APA professional |
| **Between V0 and V1: number of steps self-monitoring** | |
| **V1** | |
| Warming; Senior Fitness Test battery and static balance test; self-administered questionnaire and 24-hour diet recall. | APA professional |
| **The week before V2: number of steps self-monitoring and 24-hour diet recall at home** | |
| **V2** | |
| 24-hour diet recall and self-administered questionnaire (including FFQ); static balance test; activity on waste recycling. | Dietician |
| **The week before V3: number of steps self-monitoring and 24-hour diet recall at home** | |
| **V3** | |
| Feedback on the last 3 months; warming; Senior Fitness Test battery and static balance test; 24-hour diet recall and self-administered questionnaire (including FFQ). | APA professional |

^a^ Each visit lasted 2h30.

APA: adapted physical activity; FFQ: food frequency questionnaire; V0-V3: measurement visits.
