# Supplementary material for: Study protocol for a pragmatic cluster randomized controlled trial to improve dietary diversity and physical fitness among older people who live at home (the “ALAPAGE study”)
Source: BMC Geriatr. 2022 Aug 4;22:643. doi: 10.1186/s12877-022-03260-8 (PMC9351201; doi:10.1186/s12877-022-03260-8)
Supplement: Supplementary file 3 — Additional file 3. Diversity ALAPAGE Score: calculation method. [file 12877_2022_3260_MOESM3_ESM.docx]

**Additional file 3.** Diversity ALAPAGE Score: calculation method

Dietary diversity is assessed using the Diversity ALAPAGE Score (DAS). Specifically developed for the ALAPAGE project [1], the DAS measures an healthy dietary diversity by awarding points (positive or negative ones) depending on the number of consumption occurrences (based on one Food Frequency Questionnaire [FFQ] and two 24-hour diet recalls) of 20 ‘ALAPAGE food categories’ (see list in Table A below). The direction (positive or negative) and the number of points allocated to each ALAPAGE food category (see details in Table B below) are in line with the latest French food-based dietary guidelines [2]. The DAS for one participant is calculated by summing all attributed points.

Calculation of consumption occurrences based on the FFQ

Data from the FFQ are used to assess the number of consumption occurrences for 7 ALAPAGE food categories for which recommendations are made for a week or which are considered as rarely consumed among the French population (Table B). Moreover, for those food categories, data from the 24-hour diet recalls may have underestimated consumption (e.g., for ‘Eggs’ or ‘Legumes’).

For the ‘Oils’ category, score is not based on occurrences but estimated based on the number of different oils usually used in one week.

Calculation of consumption occurrences based on the 24-hour diet recalls

Data from the two 24-hour diet recalls are used to assess the number of consumption occurrences for 13 ALAPAGE food categories included in the DAS.

First, mixed dishes are disaggregated into ingredients using recipes, and the main ALAPAGE food categories consumed from mixed dishes are identified. Before calculating the consumption occurrences, the low consumption of an individual foods or an ALAPAGE category from mixed dishes are excluded. For individual foods, the consumption over a day less than the equivalent of a half portion, based on the standard size of portion (in g.) for each category (Table B) are excluded. For ALAPAGE categories from mixed dishes, the consumption over a meal of less than the equivalent of a half portion are excluded.

Then, for each participant, each declaration of an individual food consumption or an ALAPAGE category from mixed dishes at an eating occasion is considered as one occurrence.

Finally, for each participant, the total number of occurrences of each of the 20 ALAPAGE categories is summed.

**References**

[1] Prat R, Gazan R, Jacquemot AF, Dubois C, Féart C, Darmon N, et al. Evaluation de la validité du score de diversité ALAPAGE avec des indicateurs de qualité nutritionnelle de l’alimentation de seniors en France (INCA3). Journées Francophones de Nutrition Livre des abstracts. 2021.

[2] Ministère des Solidarités et de la Santé. Programme National Nutrition Santé 2019-2023. https://solidarites-sante.gouv.fr/IMG/pdf/pnns4_2019-2023.pdf

**Table A.** **Categorization of food into ALAPAGE families and categories**

| **ALAPAGE family** | **ALAPAGE category** | **Specific cases** |
| --- | --- | --- |
| Meats and deli meat | Poultry (and rabbits) | Addition of games in this category |
|  | Meat excluding poultry | / |
|  | Cooked ham | Only foods corresponding to "cooked ham"from this considered (including "cooked ham with rind") |
|  | Deli meat excluding cooked ham | Exclusion of foods corresponding to the "Cooked ham" category |
| Eggs | Eggs | "Egg in jelly with ham", "tortilla" were considered using recipes |
| Fishery products | Fatty fish | Foods with a content in epa/dha ≥ 1.4 g/100g and those with a content in EPA/DHA ≤1,4g/100g and a content in vitaminD ≥ 7μg/100g |
|  | Lean fish and shellfish | Foods with a content in EPA/DHA<1,4g/100g and a content in vitaminD<7μg/100g |
| Dairy products | Milk and fresh-milk dairy | Following food : "condensed milk" -> switched to "Sweetened products (including sugar)" category  Addition of chocolate drink with milk |
|  | Cheese | / |
| Legumes and nuts | Legumes | / |
|  | Nuts | Following foods: "stuffed olive", "olive n.s", "black olive", "green olive" and "violet olive" -> switched to "Salted aperitif products"  Following foods: "Mix of dried fruits of "apéritifruit" type" -> switched into "Fruits" category |
| Starches | Refined starches (including bread) and potatoes | Foods with a fibre content ≥ 5,5g/100g or ≥ 3g/100kcal -> switched into the "Semi- or wholemeal cereal products (including bread)" category |
|  |  | Only food: "wheat flake" (low sugar and fibre content) was considered |
|  |  | Foods with a fibre content ≥ 5,5g -> switched into the "Semi- or wholemeal cereal products (including bread)" category |
|  | Semi- or wholemeal cereal products (including bread) | "oat flake" and "spelt flake" (low sugar content and high fibre content) were considered in this group |
| Vegetables | Vegetables | "Spinach with cream", "Greek-style mushrooms" from the INCA3 group were considered using recipes |
| Fruits | Fruits | Syrup fruits and stewed fruit was considered in this group |
| Fats | Oils | Following foods: "oat cream", "cook soja", "fat to fry n.s", "fat to fry "vegetaline" type n.s" , "fat for cooking n.s", "fat for cooking" -> switched to "Butter, margarine, fresh cream" category |
|  | Butter, margarine, fresh cream | Following foods: "lard", "duck fat", "goose fat", "animal fat excluding butter n.s" maintained in this category |
| Products high in fats, salt and sugar | Salted aperitif products | "banana chips", "shrimp chips","carrot chips","beetroot chips" (cf sodium content) were considered in this group |
|  | Sweetened products (including sugar) | Some foods with low sugar content -> switched to "Refined starches" or "Semi- or wholemeal cereal products (including bread)" (cf particular cases in those sections)  Exclusion of "aspartame" and " aspartame-acesulfame" |
|  |  | Exclusion of drinks corresponding to "light drinks"  After analysis of the content in sugar, all juices are including in the "Sweetened drinks"  "chocolate drinks without milk" group  was considered in this group |
|  | Sweetened drinks  (including juice) |  |

**Table B.** **Allocation of score points for each ALAPAGE food category**

| **ALAPAGE family** | **ALAPAGE category** | **Method of calculation of occurrences** | **Scoring** |
| --- | --- | --- | --- |
| Meats and deli meat | Poultry (and rabbits) | Weekly frequency | Positive, in occurrence (+1 per weekly frequency) |
|  | Meat excluding poultry | Total number of occurrences on two 24H recalls | Positive if 1 occurrence and penalize over 1 occurrence (+1 if occ<=1; -1 per occ >1) |
|  | Cooked ham | Total number of occurrences on two 24H recalls | Positive if 1 occurrence and penalize over 1 occurrence (+1 if occ<=1; -1 per occ >1) |
|  | Deli meat excluding cooked ham | Total number of occurrences on two 24H recalls | Positive if 1 occurrence and penalize over 1 occurrence (+1 if occ<=1; -2 per occ >1) |
| Eggs | Eggs | Weekly frequency | Positive, in occurrence (+1 per weekly frequency) |
| Fishery products | Fatty fish | Weekly frequency | Positive, in occurrence (+2 per weekly frequency) |
|  | Lean fish and shellfish | Weekly frequency | Positive, in occurrence (+2 per weekly frequency) |
| Dairy products | Milk and fresh-milk dairy | Total number of occurrences on two 24H recalls | Positive, in occurrence (+1 per occ.) |
|  | Cheese | Total number of occurrences on two 24H recalls | Positive, in occurrence (+1 per occ.) |
| Legumes and nuts | Legumes | Weekly frequency | Positive, in occurrence (+2 per weekly frequency) |
|  | Nuts | Weekly frequency | Positive, in occurrence (+2 per weekly frequency) |
| Starches | Refined starches (including bread) and potatoes | Total number of occurrences on two 24H recalls | Positive, in occurrence (+1 per occ.) |
|  | Semi- or wholemeal cereal products (including bread) | Weekly frequency | Positive, in occurrence (+2 per weekly frequency) |
| Vegetables | Vegetables | Total number of occurrences on two 24H recalls | Positive, in occurrence (+2 per occ.) |
| Fruits | Fruits | Total number of occurrences on two 24H recalls | Positive, in occurrence (+2 per occ.) |
| Fats | Oils | Total number of different oils used in one week | Positive (+1 for each different oil ; +2 for "mixed oils") |
|  | Butter, margarine, fresh cream | Total number of occurrences on two 24H recalls | Positive to 2 occurrences and penalize over 2 occurrences (+1 if occ<=2; -2 per occ >2) |
| Products high in fats, salt and sugar | Salted aperitif products | Total number of occurrences on two 24H recalls | Positive to 2 occurrences and penalize over 2 occurrences (+1 if occ<=2 ; -2 per occ >2) |
|  | Sweetened products (including sugar) | Total number of occurrences on two 24H recalls recall | Positive to 6 occurrences and penalize over 6 occurrences (+1 if occ<=6; -2 per occ>6) |
|  | Sweetened drinks (including juice) | Total number of occurrences on two 24H recalls | Positive to 2 occurrences and penalize over 2 occurrences (+1 if occ<=2; -2 per occ >2) |

* ENNS: Etude Nationale Nutrition Santé (National Health and Nutrition Study from the French Public Health Agency); GEMRCN: Groupement d’Etude des Marchés en Restauration Collective et de Nutrition.
