# Supplementary material for: Study protocol for a pragmatic cluster randomized controlled trial to improve dietary diversity and physical fitness among older people who live at home (the “ALAPAGE study”)
Source: BMC Geriatr. 2022 Aug 4;22:643. doi: 10.1186/s12877-022-03260-8 (PMC9351201; doi:10.1186/s12877-022-03260-8)
Supplement: Supplementary file 4 — Additional file 4. Food Frequency Questionnaire (FFQ). [file 12877_2022_3260_MOESM4_ESM.docx]

**Additional file 4.** Food Frequency Questionnaire (FFQ)

1. Do you usually eat eggs every week?

*Do not count the eggs in the cakes.*

🞎 No

🞎 Yes

If yes, how many eggs do you eat per week?: ____

1. Do you usually eat poultry (or rabbit) every week?

*Examples of poultry and rabbit: chicken, turkey, duck, guinea fowl, rabbit...*

🞎 No

🞎 Yes

If yes, how many times do you eat them per week?: ____

1. Do you usually eat fatty fish every week?

*Examples of fatty fish: mackerel, sardines, herring, salmon, anchovies...*

🞎 No

🞎 Yes

If yes, how many times do you eat them per week?: ____

1. Do you usually eat lean fish or shellfish every week?

*Examples of lean fish and shellfish: cod, sea bass, sea bream, shrimp,*

*crab, mussel, oyster, squid...*

🞎 No

🞎 Yes

If yes, how many times do you eat them per week?: ____

1. Do you usually eat legumes every week?

*Examples of legumes: lentils, chickpeas, dried beans...*

🞎 No

🞎 Yes

If yes, how many times do you eat them per week?: ____

1. Do you usually eat nuts (unsalted) every week?

*Examples of nuts (unsalted): walnuts, hazelnuts, almonds...*

🞎 No

🞎 Yes

If yes, how many times do you eat them per week?: ____

1. Do you usually eat semi or wholemeal bread (or rusks) every week?

*Examples of semi or wholemeal bread (or rusks): semi or wholemeal bread, cereal bread, rye bread, wholemeal rusks...*

🞎 No

🞎 Yes

If yes, how many times do you eat them per week?: ____

1. Do you usually eat semi or wholemeal cereal products every week?

*Examples of semi or wholemeal cereal products: semi or wholemeal pasta, semi or wholemeal rice, semi or wholemeal semolina...*

🞎 No

🞎 Yes

If yes, how many times do you eat them per week?: ____

Which oils do you usually use?

*Tick the oils you use:*

🞎 Olive oil

🞎 Sunflower oil

🞎 Rape seed oil

🞎 Mix of oils

🞎 Walnut oil

🞎 Other oils

If “other oils”, which ones: _______________________________
