# Supplementary material for: Study protocol for a pragmatic cluster randomized controlled trial to improve dietary diversity and physical fitness among older people who live at home (the “ALAPAGE study”)
Source: BMC Geriatr. 2022 Aug 4;22:643. doi: 10.1186/s12877-022-03260-8 (PMC9351201; doi:10.1186/s12877-022-03260-8)
Supplement: Supplementary file 5 — Additional file 5. Self-administered questionnaires’ sections related to process evaluation. [file 12877_2022_3260_MOESM5_ESM.docx]

**Additional file 5.** Self-administered questionnaires’ sections related to process evaluation

1. At the beginning of the workshop, we gave you a letter so that you could inform the doctor that you were attending this workshop, if you wanted to. Did you give this letter to your doctor?

🞎 Yes

🞎 No

*If yes:*

- 1. What did your doctor tell you?

🞎 He told you it is good for you to participate

🞎 He told you that it is good for you to participate while taking some precautions

🞎 He did not tell you anything in particular

🞎 He rather advised you against participating

🞎 Other: ______________________________________________________

1. During the physical activity sessions, the physical activity professional showed you exercises that you can do in your daily life (for example, squats while putting something away in a cupboard). What do you think about these exercises?

|  | Strongly agree | Rather agree | Rather disagree | Strongly disagree | Don’t know |
| --- | --- | --- | --- | --- | --- |
| The exercises to be performed are too difficult |  |  |  |  |  |
| I get bored when I do the exercises (too repetitive, not repetitive, not fun) |  |  |  |  |  |
| I have the impression that the exercises are not not effective in my case |  |  |  |  |  |

1. In the past 3 months, you have done this type of exercise:

🞎 Several times a day

🞎 Once a day

🞎 Several times a week but not every day

🞎 Less frequently

🞎 Never

1. During the physical activity sessions, the physical activity professional also showed you a small exercise that you can do at home every day for about ten minutes ("the daily parenthesis"). What do you think of this "parenthesis"?

|  | Strongly agree | Rather agree | Rather disagree | Strongly disagree | Don’t know |
| --- | --- | --- | --- | --- | --- |
| The exercises to be performed are too difficult |  |  |  |  |  |
| I get bored when I do the exercises (too repetitive, not repetitive, not fun) |  |  |  |  |  |
| I have the impression that the exercises are not not effective in my case |  |  |  |  |  |

1. In the past 3 months, you have done this "parenthesis":

🞎 Every day

🞎 Several times a week

🞎 Once a week

🞎 Less frequently

🞎 Never

1. At the workshop, the dietitian gave you a "dietary diversity magnet". In the past 3 months, you have used this magnet:

🞎 Every day

🞎 Several times a week

🞎 Once a week

🞎 Less frequently

🞎 Never

1. Following my participation in the ALAPAGE workshop, I have improved my dietary diversity?

🞎 Strongly agree

🞎 Agree

🞎 Rather agree

🞎 Indifferent

🞎 Rather disagree

🞎 Disagree

🞎 Strongly disagree

1. On which family(ies) have you changed your eating behaviors since the beginning of the workshop?

🞎 None

🞎 Fruits

🞎 Vegetables

🞎 Starches

🞎 Fishery products

🞎 Meat

🞎 Eggs

🞎 Legumes and nuts

🞎 Dairy products

🞎 Fats

🞎 Products high in fats, salt and sugar

🞎 Water

1. In the past 3 months, have you done any activities with other workshop participants (walking, outings, meals...)?

🞎 Every week

🞎 Every month

🞎 Less frequently

🞎 Never

1. In the last 3 months, have you joined a club or association to practice a regular physical activity?

🞎 Yes

🞎 No

**Theory of Planned Behavior questionnaire^[[1]](#footnote-1)^**

1. I will try to do more daily physical activity in the next 3 months.

| Strongly disagree | 🞎 | 🞎 | 🞎 | 🞎 | Strongly agree |
| --- | --- | --- | --- | --- | --- |

1. For me, doing daily physical activity in the next 3 months would be…

| a. | Very bad | 🞎 | 🞎 | 🞎 | 🞎 | Very good |
| --- | --- | --- | --- | --- | --- | --- |
| b. | Not important | 🞎 | 🞎 | 🞎 | 🞎 | Very important |
| c. | Very unpleasant | 🞎 | 🞎 | 🞎 | 🞎 | Very pleasant |
| d. | Very stressful | 🞎 | 🞎 | 🞎 | 🞎 | Very relaxing |
| e. | Very useless | 🞎 | 🞎 | 🞎 | 🞎 | Very useful |
| f. | Very harmful | 🞎 | 🞎 | 🞎 | 🞎 | Very beneficial |
| g. | Very absurd | 🞎 | 🞎 | 🞎 | 🞎 | Very intelligent |

1. The majority of the people important to me think that I should do daily physical activity in the next 3 months.

| Strongly disagree | 🞎 | 🞎 | 🞎 | 🞎 | Strongly agree |
| --- | --- | --- | --- | --- | --- |

1. The majority of the people important to me want me to do daily physical activity in the next 3 months.

| Strongly disagree | 🞎 | 🞎 | 🞎 | 🞎 | Strongly agree |
| --- | --- | --- | --- | --- | --- |

1. I believe I am capable of doing daily physical activity in the next 3 months.

| Strongly disagree | 🞎 | 🞎 | 🞎 | 🞎 | Strongly agree |
| --- | --- | --- | --- | --- | --- |

1. To be fit, I need to do daily physical activity in the next 3 months, according to my needs.

| Strongly disagree | 🞎 | 🞎 | 🞎 | 🞎 | Strongly agree |
| --- | --- | --- | --- | --- | --- |

1. I will make an effort to increase my daily physical activity in the next 3 months.

| Strongly disagree | 🞎 | 🞎 | 🞎 | 🞎 | Strongly agree |
| --- | --- | --- | --- | --- | --- |

1. I am motivated to do daily physical activity in the next 3 months because it is what the majority of the people important to me expect.

| Strongly disagree | 🞎 | 🞎 | 🞎 | 🞎 | Strongly agree |
| --- | --- | --- | --- | --- | --- |

1. It depends entirely on me whether I do daily physical activity in the next 3 months.

| Strongly disagree | 🞎 | 🞎 | 🞎 | 🞎 | Strongly agree |
| --- | --- | --- | --- | --- | --- |

1. To be fit, I need to be physically active in my daily life in the next 3 months, in different ways.

| Strongly disagree | 🞎 | 🞎 | 🞎 | 🞎 | Strongly agree |
| --- | --- | --- | --- | --- | --- |

1. How much control do you believe you have to do daily physical activity in the next 3 months?

| Low | 🞎 | 🞎 | 🞎 | 🞎 | Huge |
| --- | --- | --- | --- | --- | --- |

1. If I wanted, I could do daily physical activity in the next 3 months.

| Strongly disagree | 🞎 | 🞎 | 🞎 | 🞎 | Strongly agree |
| --- | --- | --- | --- | --- | --- |

1. I am thinking of doing daily physical activity in the next 3 months.

| Strongly disagree | 🞎 | 🞎 | 🞎 | 🞎 | Strongly agree |
| --- | --- | --- | --- | --- | --- |

1. The majority of people important to me expect me to do daily physical activity in the next three months.

| Strongly disagree | 🞎 | 🞎 | 🞎 | 🞎 | Strongly agree |
| --- | --- | --- | --- | --- | --- |

1. Adapted from: González ST et al. Development and Validation of the Theory of Planned Behavior Questionnaire in Physical Activity. Span J Psychol. 2012;15:801–16; and Stolte E. et al. The Theory of Planned Behavior and Physical Activity Change: Outcomes of the Aging Well and Healthily Intervention Program for Older Adults. J Aging Phys Act . 2017 Jul;25(3):438-445. [↑](#footnote-ref-1)
