# Supplementary material for: Study protocol for a pragmatic cluster randomized controlled trial to improve dietary diversity and physical fitness among older people who live at home (the “ALAPAGE study”)
Source: BMC Geriatr. 2022 Aug 4;22:643. doi: 10.1186/s12877-022-03260-8 (PMC9351201; doi:10.1186/s12877-022-03260-8)
Supplement: Supplementary file 6 — Additional file 6. Self-administered questionnaires’ section related to participant’s characteristics. [file 12877_2022_3260_MOESM6_ESM.docx]

**Additional file 6.** Self-administered questionnaires’ section related to participant’s characteristics

1. You are:

🞎 A woman

🞎 A man

1. How old are you?

|______| years

1. Do you live:

🞎 Alone

🞎 With your spouse or others

1. Do you receive home help from a professional?

🞎 No

🞎 Yes

1. Currently, would you say that in your household, financially…

🞎 You are living comfortably

🞎 It is going well

🞎 You are getting by

🞎 You are finding it difficult

🞎 It is impossible without debt (or using consumer credit)

1. What is your highest diploma level?

🞎 No diploma

🞎 Certificate of primary studies, middle school level certificate, CAP, BEP

🞎 General, technological or vocational baccalaureate, vocational or other baccalaureate level diploma

🞎 Diploma above baccalaureate level

🞎 Other: ___________________________________________________

1. Whom can you really count on when you need help (except yourself)?

*Indicate the initials (or the first letter of the first name) of the person and the relationship you have with him/her. Each number must correspond to a single person. You may list up to 9 people. If you do not receive support, indicate "no one".*

1:__________________ 4: __________________ 7: __________________ 2:__________________ 5: __________________ 8: __________________ 3:__________________ 6: __________________ 9: __________________

1. Do you participate in activities (sports clubs, arts clubs…)?

🞎 Yes, at least once a week

🞎 Yes, at least once a month

🞎 Yes, more rarely

🞎 No, never

1. Overall, do you feel comfortable with the internet?

🞎 Not comfortable at all

🞎 Rather uncomfortable

🞎 Rather comfortable

🞎 Very comfortable

🞎 You do not use the internet

1. In the past 12 months, have you ever fallen?

🞎 Yes

🞎 No

*If yes*

- 1. How many times?

|_____|

1. Are you afraid of falling?

🞎 Yes

🞎 No

🞎 Don’t know

*If yes:*

- 1. Do you limit your movements or daily activities for fear of falling?

🞎 Yes

🞎 No

🞎 Don’t know

1. As an adult, have you played a club sport for at least 2 years?

🞎 Yes

🞎 No

🞎 Don’t know

*If yes:*

- 1. What type(s) of sport(s) did you practice in club?

*Several possible answers*

🞎 Individual sport

🞎 Team sport

🞎 Other: ________________________

1. In the past two years, have you participated in a prevention workshop…
2. … on diet?

🞎 No

🞎 Yes

1. … on physical activity?

🞎 No

🞎 Yes
