# Supplementary material for: Study protocol for a pragmatic cluster randomized controlled trial to improve dietary diversity and physical fitness among older people who live at home (the “ALAPAGE study”)
Source: BMC Geriatr. 2022 Aug 4;22:643. doi: 10.1186/s12877-022-03260-8 (PMC9351201; doi:10.1186/s12877-022-03260-8)
Supplement: Supplementary file 7 — Additional file 7. Sample size calculation. [file 12877_2022_3260_MOESM7_ESM.docx]

**Additional file 7.** Sample size calculation

We evaluate the impact of the intervention using mixed regression analyses taking into account the repeated nature of the data (see Equation 1 below). Estimation of the interaction term $\beta_{interaction}$ allows quantifying the impact of the intervention (differential effect of the variable “Time” according to the values of the variable “group”.

Equation 1:

$y_{it}=\beta_{0}+\mu_{i}+ \beta_{j}*X_{ij}+ \beta_{time}*Time$ + $\beta_{group}*Group$+$\beta_{interaction}*Time* Group+\varepsilon_{it}$

With:

$y_{it}$ = expected mean value of the dependent variable for the individual *i* at measurement time *t*

$\beta_{0}$ = constant of the model

$\mu_{i}\sim N(0,\sigma_{u})$ random constant of the model (intra-individual correlations)

Time: binary variable indicating time of measurement

Group: binary variable (intervention / control group)

$\varepsilon_{it}$: error term of the model

$X_{ij}$: vector of adjustment variables (e.g., age, sex).

Statistical power calculation is based on simulations of data and evaluation of the impact of the intervention using significance of the interaction term $\beta_{interaction}$ (Equation 1). Hypothesis are: dropout rate of 30% between T0 and T2 in both groups; similar participants’ characteristics in both groups; α = 0.05; 10,000 samples per simulation. We perform tests using different sample sizes at T0 (600 participants in the intervention group / 300 in the control group; 400 / 200; 300 / 150; 200 / 100).
